# Evaluation of Serum Supplementation on the Development of *Haemonchus contortus* Larvae In Vitro and on Compound Screening Results

**DOI:** 10.3390/ijms26031118

**Published:** 2025-01-28

**Authors:** Sandani S. Thilakarathne, Aya C. Taki, Tao Wang, Cameron Nowell, Bill C. H. Chang, Robin B. Gasser

**Affiliations:** 1 Department of Veterinary Biosciences, Melbourne Veterinary School, Faculty of Science, The University of Melbourne, Parkville, VIC 3010, Australia; sandanit@student.unimelb.edu.au (S.S.T.); aya.taki@unimelb.edu.au (A.C.T.); tao.wang1@unimelb.edu.au (T.W.); billc@unimelb.edu.au (B.C.H.C.); 2 Microscopy and Flow Cytometry Facility, Drug Discovery Biology, Monash Institute of Pharmaceutical Sciences, Monash University, Parkville, VIC 3052, Australia; cameron.nowell@monash.edu

**Keywords:** *Haemonchus contortus*, parasitic nematode, in vitro culture, serum supplementation, anthelmintic screening, phenotypic assay

## Abstract

A high-throughput platform for assessing the activity of synthetic or natural compounds on the motility and development of *Haemonchus contortus* larvae has been established for identifying new anthelmintic compounds active against strongylid nematodes. This study evaluated the impact of serum supplementation on larval development, motility and survival in vitro and its implications for phenotypic compound screening. Of five blood components assessed, 7.5% sheep serum significantly enhanced larval development, motility and survival compared to the original medium (LB*), leading to the formulation of an improved medium (LBS*). Proteomic analysis revealed marked differences in protein expression in larvae cultured in LBS* versus LB*, including molecules associated with structural integrity and metabolic processes. The phenotypic screening of 240 compounds (“*Global Priority Box*” from Medicines Malaria Venture) using LBS* yielded results distinct from those in LB*, highlighting the effect of culture conditions on screening assessments. These findings indicate/emphasise the critical need to evaluate and optimise culture media for physiologically relevant conditions in screening platforms, improving the reliability of anthelmintic discovery.

## 1. Introduction

Parasitic worms, particularly nematodes (order Strongylida), pose serious and chronic health problems in humans and animals worldwide. They infect more than one billion people globally, causing some of the most prevalent neglected tropical diseases (NTD) in tropical and subtropical regions [1,2,3]. In animals, particularly livestock, nematodes are the leading cause of anaemia, anorexia and significant production losses estimated at tens of billions of dollars per year (e.g., [4]). A prominent example of such nematodes is the barber’s pole worm, *Haemonchus contortus*, which is an economically important haematophagous gastric nematode of small ruminants, including sheep and goats [5]. The life cycle of *H. contortus* includes free-living stages (L1, L2 and L3—three larval stages) in the environment and parasitic stages (dioecious L4 and adult stages) in the host animal [6]. In brief, eggs are excreted into the environment via host faeces. The first-stage larvae (L1s) develop inside the eggs and are released within one day. Following two successive moults, they develop into third-stage infective L3s in approximately a week. The infective L3s are then ingested by the ruminant host and exsheath and develop through parasitic fourth-stage larvae (L4s) to male and female adults (within 3 weeks) in the abomasum, where they feed on blood from mucosal capillaries [6]. The adult *H. contortus* can feed up to 30–50 μL of blood per day [7].

The control of this parasite has relied largely on anthelmintic drugs regardless of the availability of a commercial vaccine, called Barbervax^®^, which is registered for use in lambs [8,9]. However, the excessive and uncontrolled use of anthelmintics has led to widespread anthelmintic resistance particularly in nematodes of the order Strongylida, including *H. contortus* [8]. Given this worldwide resistance problem and variable and short-term protection by Barbervax^®^ [9], there is a need to discover new interventions, including anthelmintics, against this and related parasitic nematodes.

Anthelmintic discovery has been based on mechanism-based and phenotypic screening approaches [10]. In mechanism-based screening, compounds are evaluated based on their ability to target specific molecules or pathways [11,12]. In phenotypic screening, also called whole-organism screening, active compounds or “hits” are identified by measuring viability or behaviours such as motility of nematodes in vitro [10,12]. This latter approach remains important for discovering new compounds with anthelmintic activity [12], and advances have enabled the rapid screening of chemical compounds against parasitic stages of *H. contortus *(reviewed by Herath et al. [13]). In 2015, our laboratory established a cost-effective method for screening whole-organism motility using exsheathed L3s (xL3s) [14]. Initially, this platform had moderate throughput capabilities, allowing ~1000 compounds to be screened weekly. Recently, a semi-automated high-throughput screening platform was developed by introducing a 384-well plate format and the use of the WMicrotracker ONE Instrument (Phylumtech, Sunchales, Santa Fe, Argentina) to measure the motility of xL3s of *H. contortus* by infrared light beam interference, making the screening process practical and rapid and allowing at least 10,000 compounds to be screened per week [15].

In addition to advancing the technical aspects of high-throughput screening, it is important to consider the culture conditions to best maintain the parasite during the screening process without introducing complexity. Although a culture medium should mimic some physiological conditions of the host animal, it must at least contain key components that support the development of the parasite consistently and reproducibly in vitro. Studies have optimised culture conditions (mimicking the host environment) such that adult stages of *H. contortus* might be raised from L3s [16,17,18]. However, a continuous life cycle in vitro is not feasible at this point. Given the challenge of maintaining worms in culture for extended periods and the need for experimental animals to produce [13], L3s and xL3s have become commonly used stages for drug screening. L3s are particularly practical because they can be stored for at least six months in a temperature-controlled incubator [14,15], and xL3s can be produced via artificial exsheathment [14] and maintained in the culture system for a period of 10 days [14].

In our high-throughput platform [15], we have been using Lysogeny broth (LB) [19] to assess the activity of synthetic or natural compounds on the motility of xL3s and the development of xL3s to L4s [15]. Although this platform has served us well and yielded valuable hits and lead candidates [14,15,20,21,22,23,24,25,26,27,28,29,30,31,32,33,34,35,36,37], we have not yet assessed larval growth/development and survival rates in vitro.

In the present study, we (i) evaluated whether the culture medium could be supplemented with one or more blood components to enhance larval development (in the transition from xL3 to L4) in vitro and maintain larval motility and survival in culture for longer periods; (ii) assessed whether the use of an “enhanced” medium improves compound screening results; and (iii) explored the protein profiles of larvae cultured under enhanced and original conditions.

## 2. Results

### 2.1. Initial Evaluation of the Supplementation of the Medium LB* with Five Individual Blood Components Showed That the Addition of 7.5% of Sheep Serum Enhanced Larval Growth/Development, Motility and Survival

First, we assessed the effect of the supplementation of LB* with each of five blood components (i.e., whole blood, red blood cells (RBCs), haemoglobin, plasma, or serum using a dilution series from 20%, 10%, 5%, 2.5% and 1.25% (*v*/*v*)) on the growth/development of xL3s (Tables S1 and S2) and showed that the addition of serum had the greatest (significant) effect. In a narrower dilution series (15.0%, 12.5%, 10.0%, 7.5% and 5.0% (*v*/*v*)), we demonstrated that the addition of 7.5% (*v*/*v*) sheep serum to LB* (=LBS*) achieved the best result at 168 h and 336 h.

**Figure 1:**
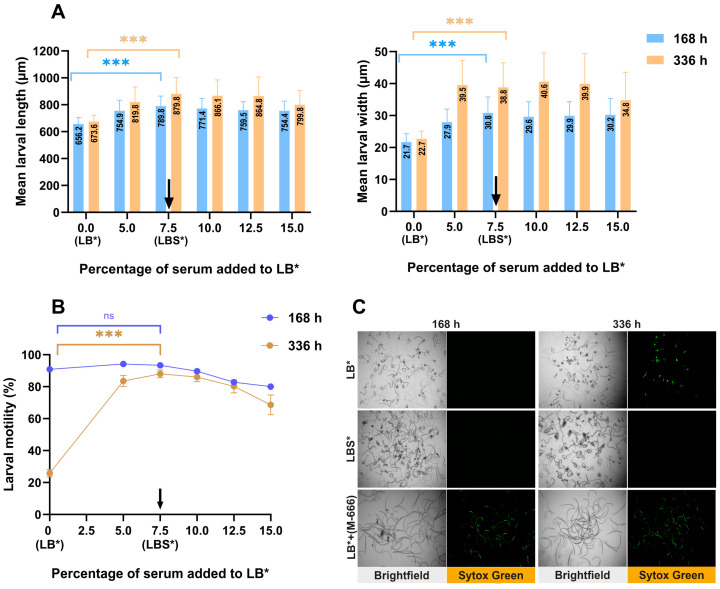
Evaluation of the growth/development, motility and survival of *H. contortus* larvae in vitro in the presence or absence of serum. (**A**) Graphs showing the mean length and width of larvae (mean ± standard deviation, SD) maintained in culture medium LB* or LBS*—data derived from two independent experiments. (**B**) Motility of larvae in LB* or LBS* measured using established methods (Section 2.3)—data points represent 18 replicates; mean ± standard error of the mean (SEM). Statistical analysis was conducted using non-parametric (Kruskal–Wallis) one-way ANOVA or Dunn’s multiple comparison test. A black arrow indicates the optimum percentage (7.5%) of serum. *** indicates a significant difference (*p* < 0.001), and ns is not significant. (**C**) Representative images of larvae in LB* or LBS* exposed to Sytox Green stain with reference to larvae exposed to the positive control compound M-666; 4-times magnification (fluorescent green = dead). M-666 likely acts on the respiratory chain and is lethal to larvae (for xL3, IC_50_ = 0. 19 µM; for L4, IC_50_ = 0.002 µM; cf. [38,39]).

**Figure 2:**
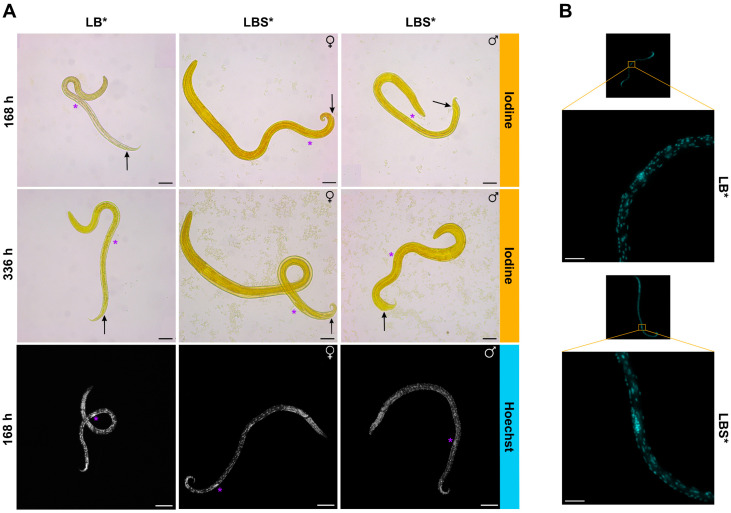
(**A**) Representative images of larvae of *Haemonchus contortus* in LB* and LBS* cultured for 168 h or 336 h and subsequently stained with iodine (10-times magnification) or Hoechst 33342 (20×/NA0.75 DRY objective). Anus (black arrow) and genital primordium (purple asterisk) are indicated (scale bar = 50 µm). Although larvae cultured in LB* did not exhibit sexual differentiation, those cultured in LBS* displayed clear dimorphism, in accordance with those originally described by Veglia [6]. In female larvae, the genital primordium was consistently positioned closer to the anus, whereas in male larvae, it was located approximately midway along the body. (**B**) Genital primordium of representative larvae in LB* or LBS* at 168 h observed using 25×/NA0.95 WATER objective (Leica Microsystems; ~70-times magnification; scale bar = 20 µm).

At 168 h, the larvae cultured in LBS* were significantly longer (mean: 789.8 ± 74.2 µm) and wider (mean: 30.8 ± 5.0 µm) than those cultured in LB* (mean: 656.2 ± 48.3 µm and 21.7 ± 2.6 µm, respectively) (Figure 1A and Figure 2A).

Larvae cultured in LB* all had a genital primordium that was located ~300 µM from the tip of the tail and comprised 6–7 cells. Larvae cultured in LBS* exhibited two distinct locations of the genital primordium (Figure 2A), comprising 11–12 cells (Figure 2B); there was an association between larval length/size and the location of this primordium. Shorter larvae (760.2 ± 62.7 µm) had a longer primordium that was more distant from the anus (~330 µm from the tip of the tail). Longer larvae (815.1 ± 75.6 µm) had a shorter primordium situated near to the anus (~180 µm from the tip of the tail). These findings appear to reflect the first stage of sexual differentiation—the former being the males and the latter the females—and are in accordance with the observations by Veglia [6].

Second, we observed that the motility of larvae in LBS* was slightly (but not significantly) higher than in LB* at 168 h but significantly higher at 336 h (Figure 1B). Third, we found that larval survival was similar between LBS* and LB* at 168 h but was significantly increased using LBS* compared to LB* at 336 h (Figure 1C). Overall, these experiments revealed that at 336 h, larval growth/development, motility and survival were significantly increased using LBS* compared to LB*.

### 2.2. Primary Screen of the ‘Global Health Priority Box’ Against H. contortus in LB* and/or LBS* and Potency Assessment In Vitro

**Figure 3:**
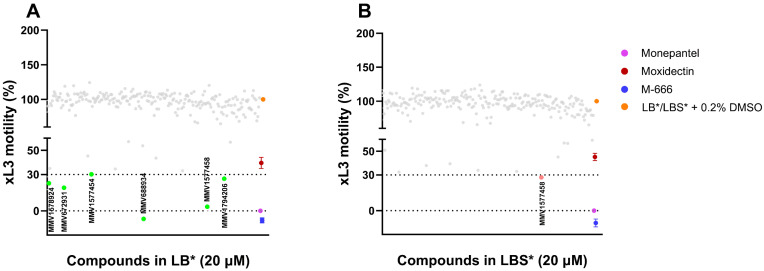
Results of the primary screen of the Global Health Priority Box compounds (n = 240) against exsheathed third-stage larvae (xL3s) of *Haemonchus contortus* using medium LB* (**A**) and LBS* (**B**). All test and positive control compounds were tested at 20 µM. Each dot represents an individual test compound. Mean ± standard error of the mean (SEM) indicated for negative and positive control compounds (eight data points for monepantel, and four for moxidectin and M-666) and negative controls (16 data points for LB* and LBS*). The Z′-factor calculated was 0.83.

Here, we explored whether the results of the in vitro screening of compounds on xL3s of *H. contortus* in LB* and LBS* would differ. Our null hypothesis was that there would be no difference. The primary screen tested 240 compounds from the Global Health Priority Box against xL3s (Figure 3).

Using LB*, 13 of the 240 compounds (Table 1) significantly inhibited the motility at 90 h and/or development at 168 h. Three of these compounds (i.e., MMV688934, MMV1577458 and MMV1794206) reduced larval motility by 74–100% and development by 100%, and each induced an abnormal phenotype: *Str* or *Cur*; Table 1; these findings were essentially consistent with previous results [36]. Three other compounds (i.e., MMV672931, MMV1578924 and MMV1577454) reduced larval motility by 71–82% at 90 h and inhibited development by 80–95% at 168 h, but none of them induced an abnormal phenotype (Table 1 and Figure 3). Seven compounds (i.e., MMV1633829, MMV1633823, MMV1633828, MMV1634081, MMV1577467, MMV002231 and MMV002519) inhibited development by ≥70% after 168 h.

Using LBS*, MMV1577458 was the only compound in the Global Health Priority Box that significantly inhibited both motility and development by 100%, and also induced abnormal (*Cur*) phenotype (Table 1 and Figure 3). Additionally, 11 compounds (i.e., MMV688934, MMV672931, MMV1578924, MMV1794206, MMV1577454, MMV1633829, MMV1633823, MMV1633828, MMV1634081, MMV1577467 and MMV002519) inhibited larval development by ≥70% at 168 h. By lowering the motility reduction threshold to ≥60%, six compounds could be identified as “hits” using LBS*. Of these six compounds, four were commonly identified as “hits” for LB* and LBS*: tolfenpyrad (MMV688934), chlorfenapyr (MMV1577458), ivermectin (MMV672931) and abamectin (MMV1577454), and two compounds were exclusive “hits” for LBS* (MMV1633829-eprinomectin and MMV1633828-moxidectin) (Table 1).

Subsequently, we estimated and compared the in vitro potencies of a set of five known compounds using LB* and LBS* (Table 2). Three of these five compounds (i.e., UMW-868, UMW-9729, ABX-464; *p* < 0.05) had significantly higher IC_50_ values when tested in LBS* than in LB* (~16.0 vs. 2.0 μM, 20.0 vs. 2.0 μM and >50 vs. 15.0 μM—see Table 2)—and two (i.e., WEHI-1617408 and M-666) did not differ significantly in their IC_50_ values between LBS* and LB* (~31.0 vs. 14.0 µM and 0.9 vs. 0.4 μM) (Table 2). The IC_50_ values for the positive control compounds monepantel and moxidectin were significantly higher in LBS* than in LB* (1.8 vs. 0.4 μM and 30.0 vs. 14.0 μM; Table 2). The IC_50_ values of four of five known compounds assessed in LB* were consistent with those reported previously (cf. [34,37,38,40]), with no value published previously for WEHI-1617408. Based on all of these findings, we rejected the null hypothesis.

### 2.3. Identification and Annotation of Differentially Expressed Proteins in H. contortus Cultured in LBS*

**Figure 4:**
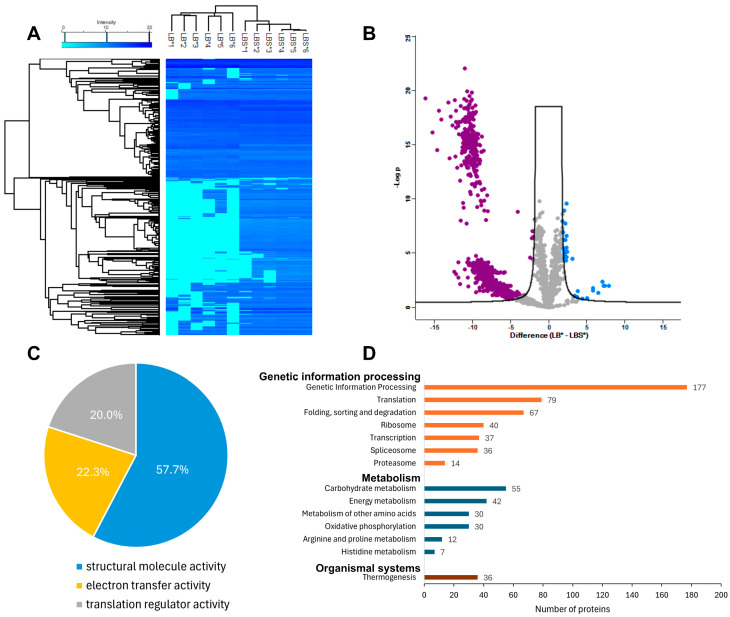
Analyses of somatic proteomes and differentially expressed proteins in the larvae of *Haemonchus contortus* cultured in medium LB* or LBS* for 168 h. (**A**) A heatmap showing relative protein abundance in larvae incubated in LB* or LBS*. Protein abundance (low to high) is shown in light to dark blue. Rows indicate individual proteins. Columns indicate six replicate samples representing larvae cultured in LB* (i.e., LB*1 to LB*6) or LBS* (LBS*1 to LBS*6). (**B**) Volcano plots of differentially expressed proteins identified between the LB* vs. LBS* (difference LB*-LBS*). Proteins that were upregulated and downregulated were in blue and purple, respectively. Proteins that were not significant are indicated in grey. Log_2_ (fold change) ≥ 2 and adjusted (*p*-value) ≤ 0.05. (**C**) The pie chart showing the distribution of the molecular functions (gene ontology (GO) level 2) of proteins quantified in larvae in LBS*. (**D**) Enriched biological processes and associated pathways (*Kyoto Encyclopedia of Genes and Genomes*, KEGG) of differentially expressed proteins in larvae LBS*. KEGG pathways sorted according to the numbers (in ascending order) of proteins in the main categories: orange (A09120—genetic information processing), blue (A09100—metabolism) and brown (A09150—organismal systems).

Somatic proteins (n = 2032) were quantified and compared between larvae cultured for 168 h in LB* vs. LBS*. A total of 1576 proteins were shared by larvae cultured in each of the media; 453 and three proteins were exclusively expressed in LBS* and LB*, respectively. Hierarchical clustering revealed two distinct protein groups expressed in the presence or absence of serum in the culture medium (Figure 4A). Volcano plot analysis revealed 1099 differentially expressed proteins (adjusted *p* ≤ 0.05), with 1069 and 30 unique to larvae cultured in LBS* and LB*, respectively (Figure 4B; Table S3).

Molecular functions of proteins uniquely expressed in LBS*-cultured larvae (n = 104) included structural molecule activity (GO:0005198; n = 75 proteins; 57.7%) and electron transfer activity (GO:0009055; 29 proteins; 22.3%) (Figure 4C; Table S4)—molecule activity related mainly to structural constituents of ribosome (n = 42 proteins) and electron transfer activity linked to proteins with oxidoreductase activity (n = 72). Biological processes inferred via pathway enrichment analysis for proteins uniquely expressed in LBS*-cultured larvae included genetic information processing (including translation, protein folding, sorting and degradation; n = 450 proteins), metabolism (carbohydrate, energy and amino acids; n = 176) and organismal systems (thermogenesis; n = 36) (Figure 4D; Table S5). The zona pellucida-domain protein (Hcon5G00000000057.2) with the highest level of significance for LBS*-cultured larvae was expressed at a >10-fold-higher level (adjusted *p* = 9.17 × 10^−23^) than in LB*-cultured larvae.

## 3. Discussion

Here, we tested different sheep blood components to assess *H. contortus* xL3 larval development in vitro. The findings showed that supplementation of culture medium with 7.5% serum had a significant positive effect on development, motility and survival.

The morphometry of *H. contortus* was used to assess larval development/growth. At 168 h (7 days), a significant difference in larval length (656.2 ± 48.3 µm vs. 789.8 ± 74.2 µm) and width (21.7 ± 2.6 µm vs. 30.8 ± 5.0 µm) was observed between LB* and LBS*. Moreover, larvae in LBS* appeared to be undergoing sexual differentiation, which was not the case for LB*. The motility and survival of larvae were similar across both media, but at 336 h (14 days), a significant increase in motility and survival was reported in LBS*. These differences are likely due to the presence of nutrients, growth factors and/or hormones in sheep serum in LBS*, required for development of larvae. The larvae in LB* were markedly less developed at 168 h (7 days) and exhibited less motility and survival at 336 h, suggesting that the larvae were deprived. In the past, some studies used blood components—including defibrinated sheep blood [42], bovine blood and calf serum [17,18], sheep or calf serum [16,43] and 10% foetal calf serum [44]—or other supplements [45,46] in culture systems for long-term culture of *H. contortus *(Table S6). Among these studies, only Stringfellow [18] succeeded in producing larvae that grew reproducibly from 400 µm to 4600 µm in length from 7 to 28 days. In the present study, larvae grew from 789.8 ± 74.2 µm to 879.8 ± 123.7 µm in length from 7 to 14 days. This discrepancy will be attributable to the different culture conditions and the strain of *H. contortus* used in each study. Some of these studies managed to grow the young adult stage in 17 days [18] and 19 days [17] and mature adults in 28 days [18] and 24–30 days [16]. Although these results are very impressive, the limitations of the cultivation systems utilised at the time are that they used complex media and methods. Here, we used a simpler medium (LBS*) and achieved initial sexual differentiation at 168 h—earlier than reported by Glaser and Stoll [42] and Niciura et al. [44] but later than reported by Veglia [6]—reaching first sexual differentiation four days after infection, with most larvae exceeding 1 mm in length. The number of nuclei within the genital primordium of individual larvae cultured in LBS* was consistent with that (n = 12) reported for the early L4 stage by Veglia [6], indicating improved development in this medium. Here, our primary focus was to enhance worm development, motility and survival in short-term culture for compound screening and further improvements to our culture medium could be made to assist longer-term maintenance of larvae in vitro.

Subsequently, we assessed whether the improved culture condition (using LBS*) would alter the results of compound screening in an established assay that uses LB*. We tested the null hypothesis that there would be no difference. All “hits” identified in LB* (13 compounds) and LBS* (12 compounds) in this study were previously identified [36]. Previous work has shown that besides a threshold of ≥70% motility reduction; two other factors were also considered to identify “hits” which inhibited L4 larval development and induced an abnormal phenotype [24,30,35,36]. Using a cut-off of ≥70% for motility reduction, six compounds in LB* versus one compound in LBS* were identified as “hits” (Figure 3). The motility reduction in LBS* was consistently lower compared to LB*, suggesting that the larvae might be more resilient and/or that the bioavailability of compounds might be limited in LBS* due to potential binding of some compounds to serum proteins or lipids. Nevertheless, lowering the motility reduction threshold to ≥60% when using LBS* allowed five additional compounds to be identified as “hits” with moderate activity, indicating the importance of considering adjusting the cut-off, depending on assay conditions. Of note is that these five compounds in LBS* induced a developmental inhibition in larvae of ≥70%.

Additionally, we carried out dose-response assays on a set of known compounds to compare the IC_50_ values of xL3 motility using LB* and LBS* employing moxidectin and monepantel as positive controls. Of five compounds, three (UMW-868, UMW-9726 and ABX-464) showed significantly different IC_50_ values. These compounds were recorded as more potent in vitro in LB* than in LBS* (2.0 vs. ~16.0 μM, 2.0 vs. 20.0 μM and 15.0 vs. >50 μM, respectively). These differences in in vitro potency may be explained by the “physiological condition or status” of *H. contortus*, depending on the medium used, with a decreased amount of available compound in LBS* due to the presence of serum and/or altered compound influx, expression level of the molecular target(s), metabolism, detoxification and/or efflux in *H. contortus *(see [47,48]). There was no difference for compounds WEHI-1617408 and M-666, suggesting that they are not absorbed by serum components and/or that they have a highly specific mechanism of action. None of the five compounds tested, except ABX-464, did not fit the Lipinski rules [41] (Table 2). Thus, the observed differences are interpreted to be due to the physiological differences in *H. contortus* larvae between the two media and/or serum absorption of some compounds. Overall, there was a significant difference in compound screening between the two media, leading to a rejection of the null hypothesis.

Proteomic analysis revealed significant distinctions in LBS*-cultured larvae, with molecular function dominated by structural molecule activity (57.7%) and electron transfer activity (22.3%). The enrichment of ribosomal proteins supports heightened protein synthesis, essential for larval growth, whereas oxidoreductase-linked proteins indicate active metabolism and redox processes. Additionally, biological processes—including genetic information processing (e.g., translation and protein folding), metabolism (carbohydrate, energy and amino acids) and thermogenesis—reflect the adaptive responses of larvae to serum supplementation, utilising nutrients for development and sustaining energy demands. The abundant expression of zona pellucida-domain protein (Hcon5G00000000057.2; Table S3) in LBS*-cultured larvae suggests its critical role in maintaining cuticle integrity and structural development. This protein, known to contribute to nematode body shape and vulval matrix composition [49,50,51], likely supports the observed sexual differentiation in larvae treated with serum. The identification of the Mago nashi protein (Hcon5G00000008116.1; Table S3), essential for regulating oogenesis and inhibiting “masculinising” genes in *Caenorhabditis elegans* (see [52]), further indicates the effect of serum in the medium on sexual differentiation in *H. contortus*. Future work might establish and compare the localisation of these proteins in *H. contortus* larvae produced in vitro and in vivo.

Serum supplementation also enhanced carbohydrate and energy metabolism, as evidenced by elevated glucose utilisation and energy production (Table S7). The enhanced expression of aspartic (A1) and metallopeptidases (M13) suggests efficient degradation of serum components, supporting larval growth and survival. Differential expression of collagens and cuticular proteins (Table S8), critical for maintaining body shape and structural integrity [53], further reinforces the positive effects of serum supplementation. These findings collectively demonstrate that serum supplementation enhances the physiological state of larvae in culture, promoting ribosomal activity, metabolic processes and structural protein expression. Thus, serum-enriched conditions support a relatively sound larval development and improve resilience.

## 4. Materials and Methods

### 4.1. Procurement of Parasite Material

The *H. contortus *(Haecon-5 strain; cf. ([54]) was produced in Merino sheep (6 months of age; male) maintained under helminth-free conditions as previously described [14,54]; animal ethics approval (permit no. 23983) was granted by the University of Melbourne in accordance with the institutional animal ethics guidelines and Australian regulations. Sheep were inoculated orally with 7000 infective L3s of *H. contortus*. After four weeks of infection, faecal samples were collected from sheep with patent infection. Faecal samples containing *H. contortus* eggs were incubated at 27 °C and >90% relative humidity for seven days to produce L3s. These larvae were suspended in tap water and sieved through two layers of nylon nitex^®^ mesh (pore size: 20 μm; Sefar, Huntingwood, New South Wales, Australia) to remove debris and dead larvae, and then stored at 11 °C for up to six months [14].

### 4.2. Procurement of Individual Ovine Blood Components

Published methods/protocols were used to prepare five different blood constitu-ents/components: (a) whole blood; the blood from a healthy parasite-free sheep was drawn from the jugular vein, collected directly into a sterile centrifuge tube (Falcon^®^, Corning, NY, USA), and mixed gently with an equal volume of Alserver’s solution (Sigma-Aldrich, MO, USA). The tube was stored at 4 °C until use (https://www.sigmaaldrich.com/AU/en/technical-documents/protocol/cell-culture-and-cell-culture-analysis/cell-based-assays/preparation-of-antibody, accessed on 10 January 2024), (b) RBCs; the whole blood in the Alserver’s solution was centrifuged at 450× *g* at 4 °C for 10 min. The RBCs at the bottom were isolated after discarding the supernatant containing plasma, buffy coat and uppermost RBCs. Then, RBCs were washed three times in sterile 0.9% saline to obtain pure RBCs [55]. The number of RBCs was counted using a hemocytometer, and LB* was added with precision to achieve the density of 1 × 10^8^ cells/mL, (c) haemoglobin (Hb); haemolysis was achieved by adding sterile water to purified RBCs in LB* [56]. The solution was centrifuged at 2000× *g* at 4 °C for 10 min to remove the cell debris, (d) plasma; blood was collected into a heparinised tube, and the plasma was separated after centrifugation at 2000× *g* at 4 °C for 15 min. Samples were then stored at −20 °C until use. The concentration of total plasma protein was assessed using the BCA Protein Assay Kit (Thermo Fisher Scientific, Waltham, MA, USA), (e) quality controlled, commercial sheep serum (cat no. 16,070,096 and lot no. 2600217, Gibco, Thermo Fisher Scientific) was purchased and stored at −20 °C until use. Protein concentrations were estimated using the BCA Protein Assay Kit.

### 4.3. In Vitro Culture Using Medium LB* Versus LBS*

The culture method originally described by Preston et al. [14] was used to assess larval growth/development, motility and/or viability of *H. contortus* from xL3 to L4. In brief, immediately prior to use in the culture assay, *H. contortus* L3s were exsheathed using 0.15% (*v*/*v*) of sodium hypochlorite (NaClO) at 38 °C for 20 min [14], achieving an exsheathment rate of 90%. Then, xL3s were rinsed five times immediately in 50 mL sterile physiological saline by centrifugation at 500× *g* for 5 min at room temperature (22–24 °C). After the last wash, xL3s were suspended in sterile lysogeny broth (LB) medium (10 g tryptone (cat. no. LP0042B; Oxoid, Basingstoke, UK), 5 g yeast extract (cat. no. LP0041; Oxoid), 5 g NaCl (ChemSupply, Gillman, SA, Australia) in 1 L of reverse-osmosis deionised water) [15,19], supplemented with 100 IU/mL of penicillin, 100 μg/mL of streptomycin and 0.25 μg/mL of amphotericin B (Fungizone^®^, cat. no. 15240-062; Gibco, Thermo Fisher Scientific)—designated LB*. The xL3s were then resuspended in LB* at a concentration of 100 xL3s per 50 µL (for assessing individual blood components) and 200 xL3s per 50 µL (for serum alone) in 96-well microplates (cat. no. 3596; Corning).

Focused on improving larval development/growth, motility and viability in vitro, we conducted a series of well-controlled, preliminary experiments to assess individual ovine blood components (i.e., whole blood, RBCs, haemoglobin, plasma and serum—assessed individually) as supplements to the original LB* medium originally used in the culture system developed by Preston et al. [14] (cf. Section 2.1). Following the addition of 50 µL of LB* with xL3s to 50 µL of each blood component, all the plates were incubated at 38 °C with a gas phase of 10% (*v*/*v*) CO_2_ and >90% humidity. The findings of these experiments demonstrated that only 7.5% (*v/v*) serum supplementation achieved a reproducible and significant enhancement in larval development, motility and viability in culture. Thus, LB* medium supplemented with 7.5% (*v*/*v*) serum, designated LBS*, achieved optimum results (detailed results presented in Section 2).

The motility of larvae cultured in LB* or LBS* was measured after 168 h and 336 h of incubation; plates were placed on an orbital shaker (model EOM5, Ratek, Boronia, VIC, Australia) rotating at speed 7 (126 rpm) for 5 min at 38 °C to agitate the larvae. Motility was recorded as a percentage of the population of each replicate using a stereo microscope (M80, Leica Microsystems, Wetzlar, Germany). The viability of larvae cultured in LB* or LBS* for 24 h (38 °C, 10% (*v*/*v*) CO_2_ and >90% humidity) in the presence of Sytox Green nuclei acid stain (Thermo Fisher Scientific) (final concentration: 1 µM) was assessed [34]. Fluorescent images of larvae in wells were captured using the EVOS M7000 Imaging System (Thermo Fisher Scientific).

### 4.4. Comparative In Vitro Screening of Compounds on H. contortus Larvae Using LB* Versus LBS*, Potency Assessment In Vitro

An established phenotypic screening assay [15] was used to test the anthelmintic activity of 240 compounds from the Global Health Priority Box [57] on *H. contortus* xL3s in medium LB* or LBS*. Individual compounds were supplied as solid samples, and each compound was reconstituted in 10 μL of dimethyl sulfoxide (DMSO; ≥99.7%, cat. no. 34869; Sigma-Adrich, St. Louis, MO, USA) to achieve a final concentration of 10 mM. Prior to screening, test compounds were each diluted to 40 μM in LB* or in LBS*. In brief, individual compounds were tested (at 20 μM; in triplicate) for their activity against xL3s (n = 80 per well) in LB* or in LBS* (384-well microplate, cat. no. 3860; Corning). Three positive control compounds—monepantel (Zolvix™; Elanco, IN, USA), moxidectin (Cydectin^®^; Virbac, Carros, France) and compound MIPS-0018666 (abbreviated herein as M-666; cf. [38])—as well as negative controls containing 0.2% (*v/v*) DMSO alone were included in each screen. The motility of larvae in individual wells was measured after 90 h and 168 h using a WMicroTracker ONE unit (PhylumTech). Over a period of 15 min, disturbance of an infrared beam in individual wells was recorded as a worm “activity count”. Activity counts were then normalised with reference to the positive (monepantel) and negative controls to remove plate-to-plate variation using the GraphPad Prism program (v.10.1.2. GraphPad Software, La Jolla, CA, USA). A compound that reduced xL3 motility by ≥70%, inhibited larval development and/or induced an abnormal phenotype (relative to the negative control) was recorded as a “hit”. Assay performance was monitored using the Z′-factor [58], calculated using data for negative (DMSO) and positive (M-666) controls on individual plates. At 168 h, larvae were fixed following the addition of 40 μL of Lugol solution (Sigma-Aldrich) to individual wells, and morphology (phenotype) recorded following microscopic examination (EVOS M7000 Imaging System (Thermo Fisher Scientific); Bright-field; 4-times magnification).

In addition, the in vitro potencies of five known compounds (UMW-868, UMW-9729, ABX-464, WEHI-1617408 and M-666); Refs. [27,34,37,38,40] were assessed on xL3s in a well-established dose-response assay [15] employing LB* or LBS* after 90 h of incubation.

### 4.5. Microscopy

Light microscopy was used to assess the morphology and morphometrics of larvae cultured in LB* in the presence or absence of whole blood, RBCs, haemoglobin, plasma, or serum for 168 h and 336 h; 10 larvae per each replicate per well were fixed in 25 µL of Lugol’s solution. The mouth, pharynx and tail were studied using a Leica DM1000 LED microscope with LAS X version 5.1.0.25446 (Leica Microsystems). The length and width (base of the oesophagus) of individual larvae [6] were measured using ImageJ 2.14.0 (https://imagej.net/software/fiji/, accessed on 10 March 2024).

Cultured larvae were also fixed in ethanol (final concentration: 50% (*v*/*v*)) and stored at 4 °C. Subsequently, larvae were washed twice with PBS (Gibco, Thermo Fisher Scientific) to remove sheaths and serum precipitates. Larvae were introduced into a new 96-black-walled clear bottom well plate (cat. no. 6055300; PhenoPlate™-96, Revvity, MA, USA) at 50 larvae per well (in triplicate) and stained in Hoechst 33342 (Thermo Fisher Scientific) solution (final concentration: 5 µg/mL) and incubated for 24 h at room temperature, protected from the light. Five larvae from each well were imaged using a TCS-SP8 confocal microscope (Leica Microsystems) employing a HC PL APO CS2 20×/0.75 DRY objective with a zoom-in factor of 1.25. In addition, selected individual larvae were also examined using a DMI8 confocal microscope with an HC FLUOTAR L VISIR 25×/0.95 WATER objective (Leica Microsystems). All digital images were analysed using ImageJ.

### 4.6. Proteomic and Bioinformatic Methods

Proteins were extracted from each of six replicates (two biological × three technical replicates) of larvae (~200) after incubation of 168 h in LB* and LBS* separately. First, samples were transferred into Eppendorf tubes (1.5 mL) and washed twice using ice cold-PBS at 1000× *g* for 2 min. Immediately after washing, samples were snap-frozen in liquid nitrogen and then stored at −80 °C until further analysis. Prior to extraction, samples were lyophilised in a benchtop manifold freeze-dryer for overnight. Freeze-dried samples were resuspended in 150 µL of lysis buffer (8 M urea in 100 mM triethyl ammonium bicarbonate (TEAB), pH 8.5) and ultra-sonicated (22.5 kHz) using Misonix Microson™ XL 2000 (Farmingdale, NY, USA) (5 cycles: 20 s on–40 s off) on ice. Next, each sample was mixed for 45 min at 21 °C (Eppendorf^®^ ThermoMixer^®^ C, Eppendorf AG, Hamburg, Germany) and centrifuged at 15,000× *g* for 15 min at 12 °C and the supernatants collected for analyses. Protein concentrations were measured using a BCA Protein Assay Kit.

Individual samples were desalted by ice cold (−20 °C) acetone and resuspended using lysis buffer (8 M urea in 100 mM TEAB) prior to protein in-solution digestion [59]. Each protein sample (8 µg) was reduced with 10 mM tris (2-carboxyethyl) phosphine (TCEP) at 37 °C for 45 min in a shaker, then alkylated with 55 mM iodoacetamide in the dark at room temperature for 30 min, followed by a trypsin digestion (Promega, Madison, WI, USA) at 37 °C for overnight. Then, tryptic samples were acidified with 1.0% (*v*/*v*) formic acid and purified using Oasis HLB cartridges (Waters, MA, USA). Eluted peptides were freeze-dried and resuspended in aqueous 2% (*w*/*v*) acetonitrile and 0.05% (*w*/*v*) trifluoroacetic acid (TFA) prior LC-MS/MS analysis.

Tryptic peptides were analysed using an Exploris 480 Orbitrap mass spectrometer (Thermo Fisher Scientific), equipped with an Acclaim Pepmap nano-trap column (Dinoex-C18, 100 Å, 75 µm × 2 cm) and an Acclaim Pepmap RSLC analytical column (Dinoex-C18, 100 Å, 75 µm × 50 cm). Tryptic peptides were injected into the enrichment column at an isocratic flow of 5 µL/min of 2% (*v*/*v*) CH_3_CN containing 0.05% (*v*/*v*) trifluoroacetic acid (TFA) for 6 min, applied before the enrichment column was switched in-line with the analytical column. Solvent A was (*v*/*v*) 0.1% formic acid, 95% H_2_O, 5% dimethyl sulfoxide and solvent B was (*v*/*v*) 0.1% formic acid, 95% acetonitrile, 5% dimethyl sulfoxide. The gradient was at 300 nL/min from (i) 0–6 min at 3% B; (ii) 6–95 min, 3–20% B; (iii) 95–105 min, 20–40% B; (iv) 105–110 min, 40–80% B; (v) 110–115 min, 80–80% B; (vi) 115–117 min 85–3% and equilibrated at 3% B for 10 min before injecting the next sample. The Exploris 480 Orbitrap mass spectrometer (Thermo Fisher Scientific) was operated in the data-dependent mode, whereby full MS1 spectra were acquired in a positive mode (spray voltage of 1.9 kV; source temperature of 275 °C), 120,000 mass-resolving power (at *m*/*z* 200), AGC target of 3 × 10^6^ and maximum IT time of 25 ms. The “top 3 second” acquisition method was used, and peptide ions with charge states of 2–6 and intensity thresholds of ≥5 × 10^3^ were isolated for MS/MS. The isolation window was set at 1.2 *m*/*z*, and precursors were fragmented using higher energy collisional dissociation (HCD) at a normalised collision energy of 30, a resolution of 15,000, a normalized AGC target of 75%, and an automated IT time selected. Dynamic exclusion was set at 30 s.

The proteome predicted from the genome *H. contortus *(see [60]) was annotated using the eggNOG database [61]. Protein identification was performed using the Mascot software (https://www.matrixscience.com/, accessed on 22 November 2024). For relative quantification comparisons, a protein present in ≥4 replicates under at least one condition were accepted. Differentially expressed proteins were defined as those with ≥2-fold change relative to one another, with an adjusted *p*-value of ≤0.05. Hierarchical clustering and volcano plot analysis were conducted using Perseus software (v.2.1.3.0; [62]). For differentially expressed proteins, molecular functions were assigned by gene ontology (GO) using EggNOG, and biological processes or pathways using the Kyoto Encyclopedia of Genes and Genomes (KEGG) database [63] employing the TBtool [64]. KEGG pathways were identified using a cut-off of *p* < 0.01 [65]. The mass spectrometry proteomic data have been deposited in the ProteomeXchange Consortium under the dataset identifier PXD060092, via the PRIDE [66] partner repository.

### 4.7. Statistical Analyses

Non-parametric (Kruskal–Wallis) one-way ANOVA and Dunn’s multiple comparison was applied to assess significant differences in morphometrics of larvae. The *t*-test was applied for comparative analysis of IC_50_ values determined for LB* and LBS*. Representation and data analysis were performed with GraphPad Prism v.10.1.2. Statistically significant values were *p* ≤ 0.05 and *p* ≤ 0.001.

## 5. Conclusions

This study showed that the supplementation of culture medium with 7.5% sheep serum (LBS*) significantly enhances the development, motility and survival of *Haemonchus contortus* larvae in vitro. Proteomic analysis revealed elevated expression of proteins associated with structural development, metabolic activity and genetic information processing, reflecting the physiological adaptation induced by serum supplementation. Notably, the enhanced expression of zona pellucida-domain and Mago nashi proteins suggests roles in cuticle integrity and sexual differentiation. Overall, these findings emphasise the importance of evaluating and optimising culture conditions to better mimic the host environment and to attempt to provide a physiologically relevant platform for the screening and discovery of anthelmintic compounds.

## Figures and Tables

**Table 1:** Results of the primary screen of the Global Health Priority Box compounds and three positive control compounds (monepantel, moxidectin and M-666) against exsheathed third-stage larvae (xL3s) of *Haemonchus contortus.* Data calculated from two independent assays (the mean ± standard error of the mean, SEM).

Compound Code		xL3s Larval Motility Reduction at 90 h (%; Mean ± SEM)		L4 Development Inhibition at 168 h (%; Mean ± SEM)		Abnormal Phenotype ^#^	
	Compound Name	LB*	LBS*	LB*	LBS*	LB*	LBS*
MMV688934	Tolfenpyrad	106.3 ± 2.0	66.1 ± 7.1	100	95	*Str*	*Str*
MMV1577458	Chlorfenapyr	96.3 ± 5.3	72.0 ± 12.7	100	100	*Cur*	*Cur*
MMV672931	Ivermectin	82.3 ± 7.6	67.5 ± 5.7	95	95	-	-
MMV1578924	Milbemectin	77.2 ± 5.3	18.8 ± 8.9	80	70	-	-
MMV1794206	Flufenerim	74.2 ± 6.2	54.9 ± 5.9	100	100	*Cur*	*Cur*
MMV1577454	Abamectin	71.3 ± 7.0	62.1 ± 6.5	95	95	-	-
MMV1633829	Eprinomectin	67.7 ± 14.3	67.0 ± 20.6	95	70	-	-
MMV1633823	Doramectin	66.1 ± 16.5	49.2 ± 17.5	85	85	-	-
MMV1633828	Moxidectin	65.5 ± 17.0	60.5 ± 34.2	100	80	-	-
MMV1634081	Fenoxacrim	58.2 ± 28.1	32.5 ± 30.2	98	100	*Str*	-
MMV1577467	Fenpyroximate	22.2 ± 22.6	43.4 ± 29.4	100	100	*Str*	*Cur*
MMV002231	Selamectin	11.3 ± 12.5	−5.3 ± 14.2	70	0	-	-
MMV002519	Rotenone	4.5 ± 9.1	−3.1 ± 5.3	90	90	*Str*	*Cur*
	Monepantel	100.0 ± 0.0	100.0 ± 0.0	100	100	*Coi*	*Coi*
	Moxidectin	60.4 ± 4.6	54.8 ± 3.1	100	95	-	-
	M-666	108.0 ± 1.9	110.0 ± 3.3	100	100	*Cur*	*Cur*

**^#^** *Coi*, coiled; *Cur*, curved; *Str*, straight; -, no apparent distinction from wild type.

**Table 2:** Five known compounds (UMW-868, UMW-9729, ABX-464, WEHI-1617408 and M-666; [27,34,37,38,40]) assessed separately for the potency of activity on exsheathed third-stage larvae (xL3s) of *Haemonchus contortus* using media LB* and LBS* after 90 h. Compounds showing significantly higher IC_50_ values using LBS* than LB* are marked with an asterisk (*p* ≤ 0.05). Data from three independent experiments; each compound was tested in triplicate (the mean ± standard error of the mean, SEM).

Compound Code	IC_50_ Value—Larval Motility (µM ± SEM)		Lipinski Rule of Five (1 to 5) ^#^				
	LB*	LBS*	1	2	3	4	5
UMW-868	2.0 ± 0.5	16.0 ± 3.6 *	270.4	4.5	4	0	0
UMW-9729	2.0 ± 0.4	20.0 ± 6.0 *	244.3	3.9	1	1	0
ABX-464	15.0 ± 0.9	>50.0 *	338.7	5.9	6	1	1
WEHI-1617408	14.0 ± 4.0	31.0 ± 12.0	312.2	3.7	7	0	0
M-666	0.4 ± 0.2	0.9 ± 0.4	373.0	3.4	7	1	0
Moxidectin	14.0 ± 2.5	30.0 ± 1.1 *	639.4	8.4	2	2	2
Monepantel	0.4 ± 0.1	1.8 ± 0.2*	473.1	5.9	1	1	1

**^#^** Lipinski rule-of-five [41]: 1 = molecular weight (g/mol); 2 = lipophilicity; 3 = H-bond acceptors; 4 = H-bond donors; 5 = number of rule of five violations.

## Data Availability

Data are contained within the article and the Supplementary Materials.

## Supplementary Materials

The following supporting information can be downloaded at: https://www.mdpi.com/article/10.3390/ijms26031118/s1.
