# Supplementary material for: Evaluation of Serum Supplementation on the Development of Haemonchus contortus Larvae In Vitro and on Compound Screening Results
Source: Int J Mol Sci. 2025 Jan 28;26(3):1118. doi: 10.3390/ijms26031118 (PMC11816986; doi:10.3390/ijms26031118)
Supplement: Supplementary file 1 [file ijms-26-01118-s001.zip › Supplementary materials_Tables1,2,6-8.pdf]

International Journal of
Molecular Sciences

MDPI

Table S1: Mean length and width of larvae treated with sheep blood components at different concentrations (cultured for 168 h).

	Mean length ± SD (μm)					
	**20 % (*v/v*)**	**10% (*v/v*)**	**5% (*v/v*)**	**2.5% (*v/v*)**	**1.25% (*v/v*)**	**0%**
**RBCs**	663.7 ± 60.9 (n = 23)	646.7 ± 70.0 (n = 23)	609.8 ± 54.0 (n = 23)	610.7 ± 70.8 (n = 21)	602.3 ± 51.5 (n = 24)	609.0 ± 48.2 (n = 20)
**Hb**	668.9 ± 147.1 (n = 30)	665.3 ± 46.6 (n = 24)	654.2 ± 67.0 (n = 30)	672.8 ± 47.8 (n = 30)	661.1 ± 66.5 (n = 30)	639.6 ± 63.1 (n = 30)
**Plasma**	681.3 ± 67.7* (n = 18)	714.6 ± 68.0* (n = 30)	698.9 ± 84.4* (n = 30)	707.0 ± 76.0* (n = 30)	655.5 ± 83.9 (n = 30)	597.3 ± 60.4 (n = 27)
**Serum**	742.2 ± 85.5* (n = 30)	800.6 ± 98.9* (n = 32)	727.7 ± 103.7* (n = 30)	744.0 ± 84.6* (n = 31)	682.1 ± 56.7 (n = 31)	625.4 ± 83.1 (n = 26)
	**Mean width ± SD (μm)**					
**RBCs**	23.5 ± 4.2	22.7 ± 2.7	21.1 ± 2.5	19.7 ± 2.5	18.8 ± 1.9	22.0 ± 3.1
**Hb**	22.7 ± 5.6	18.2 ± 1.4	20.2 ± 3.4	19.3 ± 2.5	19.1 ± 2.3	21.3 ± 3.6
**Plasma**	23.1 ± 4.6	23.0 ± 3.6	26.3 ± 4.1	27.5 ± 3.1*	22.5 ± 4.2	23.4 ± 4.3
**Serum**	25.3 ± 4.1*	31.3 ± 5.0*	29.4 ± 5.3*	25.9 ± 5.2*	23.6 ± 3.0	20.3 ± 2.9

Statistical analysis was performed by non- parametric (Kruskal-Wallis) one-way ANOVA and Dunn's multiple comparison test. *Indicates significant values (p < 0.05) and n indicates sample size. Each value represents one experiment conducted in triplicate; mean ± standard deviation (SD). RBCs - Red blood cells, Hb- Haemoglobin. Data from whole blood was not available as it did not support the larval development.

Table S2: Mean length and width of larvae treated with sheep serum and plasma at different concentrations (cultured for 336 h).

	Mean length ± SD (μm)					
	**20 % (*v/v*)**	**10% (*v/v*)**	**5% (*v/v*)**	**2.5% (*v/v*)**	**1.25% (*v/v*)**	**0%**
**Plasma**	920.2 ± 139.3* (n = 23)	936.0 ± 164.7* (n = 24)	949.1 ± 175.0* (n = 22)	910.8 ± 106.9* (n = 25)	867.2 ± 99.9* (n = 24)	671.1 ± 66.8 (n = 12)
**Serum**	974.3 ± 168.2* (n = 22)	987.8 ± 159.8* (n = 30)	1001 ±153.7* (n =28)	913.9 ± 115.7* (n = 30)	911.2 ± 95.6* (n = 28)	647.1 ± 77.0 (n = 19)
	**Mean width ± SD (μm)**					
**Plasma**	42.8 ± 15.7*	36.7 ± 10.9*	39.3 ± 11.6*	37.1 ± 8.7*	34.5 ± 8.4*	23.9 ± 4.9
**Serum**	45.4 ± 14.8*	42.6 ± 11.1*	40.3 ± 9.2*	38.4 ± 8.0*	35.9 ± 5.8*	21.0 ± 3.7

Statistical analysis was performed by non-parametric (Kruskal-Wallis) one-way ANOVA and Dunn's multiple comparison test. *Indicates significant values (p < 0.05) n indiates sample size. Each value represents one experiment conducted in triplicate; mean ± standard deviation (SD).

Table S3: The list of differentially expressed proteins in the proteome of L4 of *Haemonchus contortus* treated with LB* and LBS*.

Table S4: The number of quantified proteins involved in the molecular function (levels 2 and 3) in L4s of *Haemonchus contortus* in LBS* according to Gene Ontology (GO).

Table S5: Enriched biological categories, biological processes and Kyoto Encyclopedia of Genes and Genomes (KEGG) pathways of differentially expressed proteins in L4 of Haemonchus contortus in LBS* cultured for 168 h.

Table S6: Summary of previously published in vitro cultures of *Haemonchus contortus* using blood components.

Stage of *H. contortus*; time	Medium	Physical conditions	References
L3 to L4 (male and female); 3 weeks	0.5% agar in Ringer's solution, sheep-liver extract, heat-killed ground yeast, sheep blood and sheep kidney	39.5 °C pH 3.0	[42]
L3 to adult; 24-30 days	Chick embryo extract, autoclaved sheep liver extract, casein hydrolysate and sheep serum in a 2: 2: 2: 1 ratio	-	[16]
L3 to L4	BSSA supplemented with CEE_{50}, lamb or calf serum, Cystine-fortified 2% sodium caseinate, 2% sigma liver conc. (0.1 g/100 mL medium), vitamin mixture and an antibiotic mixture	37.5 °C pH 7.2-7.3	[43]
L3 to adult; 19 days	API-1 medium supplemented with Fildes' reagent	85% N_2:5% O_2: 10% CO_2 pH 6.4 - first week and pH 6.8 following	[17]
L3 to adult; 19 to 28 days	API-1 medium supplemented with Fildes' reagent and ovine gastric content	85% N_2:5% O_2: 10% CO_2 pH 6.4 - first week and pH 6.8 following	[18]
L3 to L4 (suggestive of sexual differentiation); 28 days	DMEM medium supplemented with 10% foetal calf serum, sodium pyruvate, non-essential amino acids and L-glutamine, co-culture with Caco-2 cells	37 °C and 5% CO_2 pH 7.4	[44]

BSSA – Balanced Salt Solution, API-1 medium consists of calf serum (see [45]), DMEM- Dulbecco's Modified Eagle Medium, CEE_{50}- 50% chick embryo extract, Fildes' reagent consists of a peptic digest of defibrinated bovine blood (see [46]), Caco-2 cells - human colorectal adenocarcinoma-derived intestinal epithelial cells.

Table S7: The list of differentially expressed aspartic peptidases and metallopeptidases in L4s of *Haemonchus contortus* treated with LBS*.

Protein IDs	EggNOG functional descriptions	$\log_2$ fold-change (LB* *vs.* LBS*)	Adjusted *p*-value
Hcon5G00000018629.2	Belongs to the peptidase A1 family	-10.52	7.75E-17
Hcon5G00000018101.5	Belongs to the peptidase A1 family	-8.93	5.48E-04
Hcon5G00000018100.1	Belongs to the peptidase A1 family	-8.77	2.26E-03
Hcon5G00000005164.1	Belongs to the peptidase A1 family	-8.24	2.88E-03
Hcon5G00000017981.2	Belongs to the peptidase A1 family	-6.90	1.28E-02
Hcon5G00000014584.1	Peptidase family M13	-11.84	1.94E-17
Hcon5G00000000482.1	Peptidase family M13	-9.66	3.19E-13
Hcon5G00000002267.1	Peptidase family M13	-1.85	7.66E-09
Hcon5G00000004494.1	Peptidase family M13	-2.03	3.42E-07
Hcon5G00000014588.2	Peptidase family M13	-9.34	6.29E-05
Hcon5G00000014578.1	Peptidase family M13	-10.41	7.78E-05
Hcon5G00000002268.1	Peptidase family M13	-6.37	1.09E-02

Log_2 (fold change) ≥ 2 and adjusted (p-value) ≤ 0.05.

Table S8: The list of differentially expressed collagens and cuticular proteins in L4s of *Haemonchus contortus* treated with LBS*.

Protein IDs	EggNOG functional descriptions	log_2 fold-change (LB* *vs.* LBS*)	Adjusted *p*-value
Hcon5G00000003966.1	Structural constituent of cuticle	-5.55	1.54E-01
Hcon5G00000007502.1	Structural constituent of cuticle	-5.18	1.81E-01
Hcon5G00000008482.1	Nematode cuticle collagen N-terminal domain	-13.13	1.27E-19
Hcon5G00000008716.1	Nematode cuticle collagen N-terminal domain	-7.05	1.02E-02
Hcon5G00000008810.1	Nematode cuticle collagen N-terminal domain	-14.60	2.94E-15
Hcon5G00000009164.1	Nematode cuticle collagen N-terminal domain	-10.38	1.74E-19
Hcon5G00000009165.1	Nematode cuticle collagen N-terminal domain	-5.84	7.67E-02
Hcon5G00000014117.1	Nematode cuticle collagen N-terminal domain	-7.35	7.40E-02
Hcon5G00000014119.1	Structural constituent of cuticle	-15.19	7.49E-17
Hcon5G00000014121.1	Nematode cuticle collagen N-terminal domain	-10.49	1.21E-02
Hcon5G00000014330.1	Nematode cuticle collagen N-terminal domain	-8.56	3.23E-02

Log_2 (fold change) ≥ 2 and adjusted (p-value) ≤ 0.05.
